# MiRNAs in Radiotherapy Resistance of Nasopharyngeal Carcinoma

**DOI:** 10.7150/jca.42734

**Published:** 2020-04-06

**Authors:** Yutong Tian, Lu Tang, Pin Yi, Qing Pan, Yaqian Han, Yingrui Shi, Shan Rao, Shiming Tan, Longzheng Xia, Jinguan Lin, Linda Oyang, Yanyan Tang, Jiaxin Liang, Xia Luo, Qianjin Liao, Hui Wang, Yujuan Zhou

**Affiliations:** 1 The Affiliated Cancer Hospital of Xiangya School of Medicine, Central South University and Hunan Cancer Hospital, Key Laboratory of Translational Radiation Oncology, Hunan Province, 283 Tongzipo Road, Changsha 410013, Hunan, China.; 2 University of South China, Hengyang, 421001, Hunan, China.

**Keywords:** nasopharyngeal carcinoma, radiotherapy resistance, miRNAs, apoptosis, miR-BARTs

## Abstract

Nasopharyngeal carcinoma (NPC) is one of the most common malignant tumors of the head and neck in Southeast Asia and southern China. Although the comprehensive treatment based on intensity-modulated radiation therapy improves outcomes, the five-year survival rate of NPC patients is low, and the recurrence remains high. Radiotherapy resistance is the main cause of poor prognosis in NPC patients. MicroRNAs (miRNAs) are a class of endogenous non-coding RNAs regulating various biological functions in eukaryotes. These miRNAs can regulate the development and progression of nasopharyngeal carcinoma by affecting the proliferation, apoptosis, movement, invasion and metastasis of NPC cells. The abnormal expression of miRNAs is closely related to radiotherapy sensitivity and prognosis of NPC patients, which can affect the transmission of related signaling pathways by regulating the expression of tumor suppressor genes and / or oncogenes, and therefore participate in radiotherapy resistance in nasopharyngeal carcinoma. Here, we review the mechanisms by which miRNAs may be involved in the radiotherapy resistance of nasopharyngeal carcinoma.

## Introduction

Nasopharyngeal carcinoma (NPC) is a malignant tumor originating from nasopharyngeal epithelial cells. Etiological factors include genetics, EBV infection, environment, smoking and other factors 1, 2. Nasopharyngeal carcinoma occurs mainly in Southeast Asia and southern China, and its morbidity and mortality rank first in head and neck malignant tumors 3-5. NPC is mainly poorly differentiated squamous cell carcinoma and undifferentiated carcinoma; the special anatomy and local invasive growth characteristics of NPC make it unsuitable for surgical treatment, and radiotherapy is the best choice for NPC. Although the radiotherapy is more advanced, the recurrence rate of nasopharyngeal carcinoma is still high at 82%. Radiotherapy resistance is the leading cause of local recurrence and distant metastasis 6, 7.

MicroRNAs (miRNAs) are a type of endogenous non-coding RNA with a length of about 22 nt (19-25 nt). They bind to target mRNA molecules through partial or complete complementary sequences or to specific proteins to form miRNA-induced silencing complex and inhibit the translation of target mRNAs 8. Many previous studies have found that miRNAs regulate the expression of key genes in cell cycle, apoptosis and migration, and affect the proliferation, invasion and metastasis of various tumor cells including nasopharyngeal carcinoma 9-14. Some scholars have compared microarray data of miRNA expression profiles in radiotherapy-sensitive and radiation-resistant nasopharyngeal carcinoma cells, identified differentially expressed miRNAs and mRNAs, and constructed a post-transcriptional regulatory network of more than 300 miRNA target gene pairs 11, 15. Similar studies have been reported by other scholars to show that miRNAs ectopically expressed in nasopharyngeal carcinoma cells affects the expression of their target genes or proteins, thereby affecting the relevant signaling pathways and thereby participating in radiotherapy for nasopharyngeal carcinoma 16. This article updates the differential expression of miRNA molecules in the radiotherapy resistance of nasopharyngeal carcinoma, and explores the mechanism of miRNA in the radiotherapy resistance of nasopharyngeal carcinoma.

## Mechanism of radiotherapy resistance in nasopharyngeal carcinoma

Radiation therapy (RT) is a local treatment for curing or palliative treatment of tumors. RT works through damaging the DNA strands of tumor cells to directly kill tumor cells or make them lose the ability of infinite proliferative. However, increased tumor volume, oxygen reduction, and dysregulation of various genes can lead to tolerance of tumor cells to radiation, and thus reduce sensitivity, i.e., radiotherapy resistance. The specificity of radiotherapy resistance is the decrease of apoptosis after irradiation. The reasons at the gene level include the following two types: the decrease of apoptotic genes or the increase of the expression of anti-apoptotic genes and proliferating genes, and the enhancement of genes related to DNA damage repair, or the expression of cell cycle regulatory genes is dysregulated 6, 7, 17, 18. Many studies have shown that miRNAs participate in tumor radiation resistance through the above mechanisms 19-21.

Apoptosis is the most important mechanism of radiation therapy 22. Abnormal expression of apoptosis-related genes can inhibit the apoptosis of tumor cells induced by ionizing radiation and increase their survival, thereby promoting the radiotherapy resistance of NPC. The role of genes regulating cell proliferation is opposite to that of apoptotic genes, which can promote the survival and proliferation of tumor cells. The abnormal activation and expression of cell proliferation genes is also one of the mechanisms of tumor radiotherapy resistance. There are a large number of genes involved in proliferation and apoptosis, which are mainly divided into the following families: caspase, bcl-2, fas, and the most frequently mutated p53 gene in tumors. Proliferation-related genes are mainly myc, some factors in the bcl-2 family, and so on. They participate in the regulation of apoptosis, either alone or in concert 23-26. Mutations in the p53 gene occur in nearly 50% of human malignancies, which greatly increases the cell resistance to radiation 27, 28. Studies have shown that up-regulation of Bcl-2 expression can inhibit the expression of Bax, caspase-3 and other apoptotic genes and promotes the survival of nasopharyngeal carcinoma cells, thus playing a role in the development and metastasis of nasopharyngeal carcinoma 29. Fas belongs to the TNF receptor family. Usually, when fas protein binds to its ligand, it activates caspases, which causes apoptosis of targeted cells.

Radiation directly ionizes target cell DNA or ionize water to generate free radicals that cause DNA damage in target cells, including double strained break (DSB) and single strained break (SSB). DSB mainly causes irreversible fatal injury. SSB mainly causes reversible potential lethal damage (PLD) and sub-lethal damage (SLD) under certain conditions. The hallmark of DNA double-strand break (DSB) is phosphorylation of histone H2AX in chromatin to produce γ-H2AX 30. In eukaryotes, DNA damage is repaired after irradiation through DNA damage response (DDR) 31. The repair of DNA damage greatly affects the survival of tumor cells, producing the resistance of radiotherapy.

Experiments have confirmed that the radiosensitivity of tumor cells is closely related to the distribution of tumor cell cycle phases. At different phases of the cell cycle, the ability of radiation to kill cells is different. The most sensitive to radiation in the cell cycle is the M phase, followed by the G2 phase, and the cells in the G1 and S phases are less sensitive to radiation. Because of the active DNA segregation and cell division activity in G2 and M phases, radiation can easily cause DNA damage and cell damage, leading to unprogrammed cell death. Moreover, the periodic checkpoint is also an important part of DNA damage repair. The tumor suppressor gene p53 removes DNA abnormalities through cell cycle inhibition and induction of apoptosis.

## MiRNAs affect the sensitivity of radiotherapy for nasopharyngeal carcinoma

Since miRNA was discovered in Caenorhabditis elegans, its role as a non-coding RNA in regulating various biological behaviors of animals and plants has received widespread attention, especially in oncology 32. MiRNAs have been widely reported to regulate the occurrence and development of tumors by binding to their targeted mRNAs or by interacting with other non-coding RNAs, and this is not an exception in nasopharyngeal carcinoma. Many miRNAs have been found to be differentially expressed between patients with nasopharyngeal carcinoma and normal people 33, 34. These miRNAs can be roughly divided into two types; one is encoded by human cells and the other is Epstein-Barr virus (EBV) 35. These miRNAs affect the proliferation, invasion and migration, apoptosis, therapeutic sensitivity and other biological behaviors of NPC 36-38.

Radiotherapy sensitivity of tumors is an important basis for tumor radiotherapy. Several groups of researchers have identified differentially expressed miRNAs in radiosensitivity and radiation-resistant cells of nasopharyngeal carcinoma by gene sequencing and microarray analysis. miRNA-762, miRNA-1202, is up-regulated in radiotherapy-resistant nasopharyngeal carcinoma cells. miRNA-4291, miR-19b-3p, miR-21, miRNA-23a, miR-205, miR-483, let-7e, miR-193b and miR-7, etc., more than 60, can regulate nasopharyngeal carcinoma radiation resistance. The down-regulated miRNAs includes miR-125, miR-660, miR-203, miR-130a, miR-30a and miR-23a, more than 30, which can regulate the sensitivity of radiotherapy for nasopharyngeal carcinoma. These up-regulated or down-regulated miRNAs can regulate the radiotherapy resistance of nasopharyngeal carcinoma cells by acting on different target genes and pathways 16, 39, 40. For example, miR-205 inhibits PTEN, thereby promoting AKT to increase the resistance of nasopharyngeal carcinoma cells to radiation, facilitating their survival after irradiation 41, 42. MiR-20a-5p reduces the sensitivity of nasopharyngeal carcinoma cells to radiotherapy by targeting NPAS2 and Rab27B, and miR-193a-3p enhances the anti-radiation ability of NPC by targeting SRSF2 43-45. Different miRNAs can directly reduce the sensitivity of nasopharyngeal carcinoma cells to radiation by directly or indirectly regulating different signaling pathways, but the expression and up-regulation of miRNAs that enhance radiosensitivity in nasopharyngeal carcinoma are rarely reported.

## Mechanisms of miRNAs in radiotherapy resistance in nasopharyngeal carcinoma

In this section, we mainly describe the molecular mechanisms of miRNA involved in the radiotherapy resistance of nasopharyngeal carcinoma by affecting apoptosis, DNA damage repair and cell cycle progression of nasopharyngeal carcinoma cells.

### MiRNAs participate in radiotherapy resistance of nasopharyngeal carcinoma by affecting the transmission of bcl-2 apoptosis family, Caspase and Fas family signals

MiR-19b-3p, miR-125b, miR-21, and miR-205 promote the radiotherapy resistance of nasopharyngeal carcinoma by regulating the Bcl-2 gene family protein 6. miR-185 regulates the Bcl-2 family to promote the sensitivity of nasopharyngeal carcinoma cells to radiation. Bcl-2 is an apoptosis-inhibiting gene, and at least 19 homologs have been discovered, which play a regulatory role in the mitochondrial-dependent apoptotic pathway and control the release of cytochrome c and other apoptotic factors in mitochondria 46, 47. Members of the Bcl-2 family contain 1-4 Bcl-2 homology domains (BH1-4), of which BH4 is a domain unique to anti-apoptotic proteins, and BH3 is a domain involved in promoting apoptosis. According to function and structure, the Bcl-2 gene family can be divided into two groups; one is anti-apoptosis, such as Bcl-2, Bcl-xl, Bcl-w, and Mcl-1; the other is pro-apoptosis, including: Bax, Bad, Bak, Bid, Bim, Puma, etc. NF-κB is a transcription factor of Bcl-2, which can induce the expression of anti-apoptotic genes and stimulate cell proliferation and prevent apoptosis 48. In addition, NF-κB stimulates the caspase-8 inhibitor FLIP, an inhibitor of the apoptotic proteins c-IAP1 / 2 and XIAP.

Huang, T. et al. analyzed the expression of miR-19b-3p in five nasopharyngeal carcinoma cell lines (CNE1, CNE2, 5-8F, 6-10B, and HNE1) and normal nasopharyngeal epithelia cell line NP69 by qRT-PCR and other techniques. The expression of miR-19b-3p in radiotherapy-resistant cells of nasopharyngeal carcinoma was found to be significantly higher than that in normal nasopharyngeal tissues. Moreover, miR-19b-3p can increase the expression of Bcl-2 by targeting tumor necrosis factor alpha-induced protein 3 (TNFAIP3, also known as A20), while the expression of apoptosis-promoting factor Bax is decreased in NPC tissues and cells. TNFAIP3 can increase apoptosis by inhibiting the expression of Bcl-2 transcription factor NF-κB. miR-19b-3p suppresses TNFAIP3 expression and activates NF-κB pathway, which reduces apoptosis of irradiated NPC cells. Similarly, Li, et al. found that miR-125b is up-regulated in nasopharyngeal carcinoma cell lines and promotes radiotherapy resistance of nasopharyngeal carcinoma cells by targeting the TNFAIP3/NF-κB pathway. However, miR-21 and miR-205 are highly expressed in NPC cells resistant to radiotherapy, and the radiotherapy resistance is associated with inhibition of PTEN signaling pathway 41, 42, 49. Inactivation of PTEN results in constitutive activation of the STAT3 and PI3K-AKT pathways and subsequent protein synthesis, inhibiting apoptosis. Experiments have shown that Bcl-2 protein levels are increased in miR-205 transfected CNE-2 cells, and studies have shown that STAT3 activation can inhibit cell apoptosis by blocking the expression of Bim protein 50, 51. However, miR-185, which enhances apoptosis, can increase the expression of Bax and Bid and decrease the expression of Bcl-2, promoting apoptosis, 52. All these indicate that miR-19b-3p, miR-125b, miR-21, and miR-205, which are elevated in nasopharyngeal carcinoma, promote radiotherapy resistance through the bcl-2 apoptosis family, while miR-185 causes nasopharyngeal cancer cells sensitive to radiation therapy.

The researchers found that up-regulated miR-21 and miR-BART4 or down-regulated miR-143 and miR-153 in nasopharyngeal carcinoma can inhibit the apoptosis of nasopharyngeal carcinoma cells via Fas or Caspase pathway, accompanied by or not with the regulation of Bcl-2 family proteins, and the sensitivity of nasopharyngeal carcinoma cells to radiotherapy is reduced. Wu, Q. et al. found that EBV-encoded miR-BART4 promotes proliferation and invasion of nasopharyngeal carcinoma cells in nasopharyngeal carcinoma, and promotes radioresistance of nasopharyngeal carcinoma. They confirmed by further experiments that overexpression of miR-BART4 can reduce the expression of cleaved caspase-3, inhibiting apoptosis of nasopharyngeal carcinoma cells. After 6Gy irradiation treatment, the level of caspase-3 and apoptosis in the miR-BART4 overexpressed group was significantly lower than that in the NC group and the miR-BART4 inhibitor group, and in this process, bcl-2 and bax also appeared according change 53. Chen, J. H. and other scholars have found that miR-143, which is down-regulated in nasopharyngeal carcinoma, can initiate apoptosis through caspase-3. As the amount of transfected miR-143 increases, the expression level of caspase-3 increase aacordingly. The level of bcl-2 did not change during this process 54. Similarly, miR-153 expression was significantly reduced in NPC patients compared to adjacent tissues. Up-regulation of miR-153 induces apoptosis, increases caspase-3 and caspase-9 activity, and increases B-cell lymphoma 2 (Bcl-2)-associated X protein/Bcl-2 protein expression in 13-9B cells 55.

It is well known that Fas, also known as APO-1, TNFR (tumor necrosis factor receptor) and the NGF receptor family can drive cells to pass the Caspase pathway when they bind to their receptors 56. Caspases belong to cysteine ​​proteases and are a key enzyme that causes apoptosis. Once activated, it can degrade proteins in cells and induce apoptosis. Top caspase initiation and effector caspases trigger apoptosis, and different members of the caspase family are involved in endogenous or exogenous apoptosis programs, such as caspase-9 or caspase-10, which are involved in exogenous apoptosis 57, 58. Moreover, caspases play an important role in the immune system triggered by DNA virus or RNA virus infection. What role does caspase then play in nasopharyngeal carcinoma closely related to Epstein-Barr virus? Interactions that regulate tumor apoptosis are a question worthy of exploring. In addition, the replication and high expression of EBV virus is one of the important factors in the poor prognosis of nasopharyngeal carcinoma. The level of EBV DNA is closely related to local recurrence and distant metastasis after treatment in patients with nasopharyngeal carcinoma 31, 59. EBV infection can induce the expression of a variety of miRNAs including microRNA-21, and scholars have found that Epstein-Barr virus-encoded LMP1 (latent membrane protein 1) can increase miR-21, negatively regulate pro-apoptotic factor procedural cell death 4 (PDCD4) and Fas ligand (Fas-L) increase resistance to cisplatin treatment in nasopharyngeal carcinoma cells 60. Platinum drugs combined with radiotherapy can more effectively kill hypoxic tumor cells in less solid tumors. Based on this principle and clinical practice, platinum-based synchronous chemotherapy combined with radiotherapy is defined as the standard treatment of advanced nasopharyngeal carcinoma. This indicates that the high expression of miR-21 also indirectly causes radiotherapy resistance of nasopharyngeal carcinoma 61, 62.

Among them, the genes up-regulated include miR-19b-3p, miR-125b, miR-21, miR-205, miR-BART4, miR-143, and the down-regulated genes are miR-185, miR-153.

### 4.2 miRNAs participate in radiotherapy resistance of nasopharyngeal carcinoma by regulating DNA damage repair

Although irradiated cells can be apoptotic, not all cells enter apoptosis, which is one of the causes of radiotherapy resistance. When the cells are irradiated with a non-lethal dose and the single-strand or double-strand DNA break damage is repaired, the cells may survive without apoptosis.

Zhou, X. et al. found that EBV-miR-BART8-3p promotes NPC cell proliferation after irradiation in vitro and is associated with induction of cell cycle arrest in G2/M phase. In addition, in vitro animal experiments also indicate that EBV-miR- BART8-3p significantly increased the size of xenografts in nude mice and promotes radioresistance of NPC by regulating the activity of the ATM/ATR signaling pathway 63. Studies have shown that the other four miRNAs encoded by EBV (BART5-5p, BART7-3p, BART9-3p and BART14-3p) directly target endogenous ATM, and the reduced ATM also helps maintain the virus incubation period 31, 64. MiR-24 expression was found to be decreased in nasopharyngeal carcinoma cells and patients with higher stage nasopharyngeal carcinoma, and this change was associated with the development of radiotherapy resistance 65, 66. Scholars also found that miR-24 can inhibit the repair of DNA damage by targeting Jab1, and thus increase the sensitivity of nasopharyngeal carcinoma cells to radiotherapy 66. JAB1 (c-Jun activation domain binding protein-1) is involved in the repair of DNA double-strand breaks, which maintain the stability of the genome. The deletion of Jab1 leads to spontaneous DNA fragmentation and increased expression of histone γ-H2AX 67-69.

The protein encoded by the ATM gene belongs to the PI3 / PI4 kinase family. This protein is an important cell cycle checkpoint kinase that acts as a regulator of a variety of downstream proteins, including the tumor suppressor proteins p53 and BRCA1, checkpoint kinase CHK2, checkpoint proteins RAD17 and RAD9, and the DNA repair protein NBS1. ATM participated in the cell cycle and DNA damage repair, two important processes in radiation injury 70, 71. In cells with mild DNA damage, ATM activates cell cycle checkpoints, stops cell cycle progression, and initiates DNA repair process. However, in cells with irreparable severe DNA damage, ATM triggers apoptosis to remove cells 72. Moreover, some scholars have found that severe hypoxia in solid tumors increases the expression of ATM and initiates DNA repair 64, 73.

### miRNAs participate in radiotherapy resistance of nasopharyngeal carcinoma by regulating cell cycle

In addition to DNA damage repair and apoptosis involved in nasopharyngeal carcinoma radiotherapy resistance, miRNAs also triggers the radiotherapy resistance of nasopharyngeal carcinoma by regulating the cell cycle. The cell cycle regulatory system can be easily divided into three parts, cyclins, cyclin-dependent cellular kinases (CDK), and CDK kinase inhibitors (CDKI). In mammalian cells, retinoblastoma protein (Rb) regulates the progression of G1 to S phase 74. At the G1 phase of the cell cycle, Rb binds to E2F-DP1 and inhibits downstream transcription. When the cells enter the S phase, G1/S CDK inactivates Rb, resulting in the release of Rb from E2F-DP1 and activation of the E2F target gene, thereby promoting G1 activation and transition to S phase. Because cells in different phases have different sensitivity to radiation, cell cycle distributions directly affect the radiosensitivity of nasopharyngeal carcinoma cells.

Wu, et al. found that overexpression of miR-188 blocks nasopharyngeal cancer cells in the G1 phase, through inhibiting Rb phosphorylation and down-regulates E2F transcription, and then inhibiting the mRNA and protein expression of CDK4 and CDK2, therefore enhances the radiosensitivity of nasopharyngeal carcinoma cells^75^. Juan Lu et al found that miR-26a, which is down-regulated in nasopharyngeal carcinoma cells and patients, inhibits the expression of cyclin D3, CDK4 and CDK6, but activates p14 (ARF) and p21 (CIP1) in an EZH2 (enhancer of zeste 2 polycomb repressive complex 2 subunit)-dependent manner, thus induces cell cycle arrest in G1 phase and enhances radiosensitivity of nasopharyngeal carcinoma^76^ . In addition, some scholars have found that miR-23a can make more cells in the G2-M phase, indicating that miR-23a is blocked by irradiation, and the tumor cells are not sensitive to radiation 77. In addition, serum miR-663 levels in NPC patients are significantly elevated and directly target cyclin-dependent kinase inhibitor 2A (CDKN2A), thereby promoting cell cycle progression and cell proliferation 78.

### miRNAs participate in radiotherapy resistance of nasopharyngeal carcinoma by other means

Numerous studies have shown that radiation can directly induce the production of a variety of inflammatory factors including IL-6, IL-8, IL-1α, IL-1β, IL-18, TNF-α, IFN-γ, etc. Inflammation, the tumor microenvironment of pro-oxidants, eventually leads to tumor cell death 79, 80. MiRNAs target these inflammatory factors to participate in the radiosensitivity of nasopharyngeal carcinoma. Studies have shown that both down-regulated miR-23a and miR-203 in nasopharyngeal carcinoma radiotherapy-resistant cell lines (CNE-2-IR) can target IL8 and activate Stat3, which inhibits apoptosis, or AKT that affects radiotherapy resistance in nasopharyngeal carcinoma 77, 81. Blood IL-17 was significantly elevated in NPC patients compared with normal subjects. The expression of miR-135a in cancer cells isolated from nasopharyngeal tumors was significantly lower than in NP69 cells, and inhibition of IL-17 by miR-135a mimics resulted in significant inhibition of NPC cell proliferation. These findings suggest that down-regulation of miR-135a may contribute to the development of NPC through a mechanism by which IL-17 stimulates the expression of pro-inflammatory cytokines 82. Moreover, the aforementioned NF-κB signaling pathway is also involved in the release and regulation of inflammatory molecules. In addition, IL-6 molecules have been reported to promote Stat3 and Nrf2-antioxidant pathway in oral squamous cell carcinoma, and up-regulation of Mn-SOD reduces oxidative stress leading to radiotherapy resistance 83. However, more miRNAs and inflammatory factors play a role in radiotherapy resistance and specific pathways remain to be further explored.

The stemness of tumor cells plays an important role in the growth, metastasis and drug resistance of various tumors 84. MiRNAs are also involved in the maintenance and enhancement of the stemness of tumor cells. Previous studies have found that a variety of miRNAs were involved in the maintenance of the stemness of nasopharyngeal carcinoma tumor cells, leading to tumor progression and therapy resistance. For example, Cai, et al. found that miR-BART7-3p encoded by EBV can increase the number of side population (SP) cells in nasopharyngeal carcinoma cells compared with control cells by targeting SMAD7, while the SP cells are reported to be primitive stem-like population^85^. Wang, et al. found that c-MYB strengthens the stemness of NPC cancer stem cell via down-regulating miR-143, thereby increasing the resistance to radiotherapy 86. Other articles also mentioned the effects of miRNAs on the stemness of nasopharyngeal carcinoma cells and the regulation of apoptosis and damage repair of cells by affecting stemness 87.

Local recurrence and metastasis after intensive radiotherapy are the main manifestations of radiotherapy failure. Studies have shown that miR-BART7 and miR-BART13 encoded by EBV and Epstein-Barr virus (EBV) immunoglobulin A / capsid antigen (IgA / VCA) and antigen (IgA / EA) have similar effects in predicting the recurrence and metastasis of nasopharyngeal carcinoma 88. Radiotherapy can significantly reduce miR-BART7 and miR-BART13 in the plasma of patients with nasopharyngeal carcinoma, but those patients with signs of local or regional persistent tumors or metastases have no significant decrease after radiotherapy 88. Other scholars have found that miR-150 expression is elevated in patients with nasopharyngeal carcinoma, and that the high expression of miR-150 is significantly related to tumor recurrence after radiotherapy; the target molecule FoxO4 of miR-150 is significantly reduced during this process 89. Thus miR-150 and its target molecule FoxO4 may be a predictors of nasopharyngeal carcinoma recurrence. In addition, various miRNAs have been reported as plasma biomarkers for monitoring the recurrence and metastasis of nasopharyngeal carcinoma; the abnormal expression of these miRNAs in nasopharyngeal carcinoma affects the sensitivity of nasopharyngeal carcinoma to radiotherapy and chemotherapy, leading to the recurrence and metastasis of nasopharyngeal carcinoma and ultimately to poor patient prognosis 35, 90. However, their specific mechanism needs further research and exploration.

## Prospect

Radiotherapy resistance of nasopharyngeal carcinoma causes poor local control, recurrence and distant metastasis, which is significantly associated with poor prognosis. In recent years, miRNAs have been found to be involved in the regulation of tumor development and progression by regulating target gene expression. In miRNAs associated with radiation resistance in nasopharyngeal carcinoma, up- or down-regulations of miRNAs have been found to be closely related to radioresistance. In addition to the miRNA molecules discussed above, there are still many miRNA molecules involved in radiotherapy resistance of nasopharyngeal carcinoma, such as miR-203, miR-150, miR-324-3p, miR-222, and miR-495, etc. The mechanisms of regulating the radiotherapy resistance of nasopharyngeal carcinoma by miRNAs can be roughly classified into the above-mentioned regulation of apoptosis cell cycle or DNA damage repair, but whether they function alone or jointly to regulate the nasopharyngeal radiotherapy resistance is not clear yet 81, 92-95.

By detecting the expression level of specific miRNA in tissues or serum of patients with nasopharyngeal carcinoma, the radiotherapy effect may be predicted, and the nasopharyngeal carcinoma tissues and cells are sensitive to radiotherapy by changing the content of these specific miRNAs at the pre-transcriptional level. According to its regulation of the body immunity, combined with immunotherapy, the therapeutic effect of the tumor is further enhanced so that the tumor patients get the maximal benefit 96, 97. However, miRNAs are involved directly or indirectly in the regulation of multiple pathways, and are found to play an opposite role in many tumors. The mechanism is still unclear and further research is needed. Moreover, whether there are other miRNAs involved in the regulation of radiotherapy resistance of nasopharyngeal carcinoma and whether other mechanisms and pathways are involved, extensive research and discovery are still needed.

## Figures and Tables

**Figure 1:**
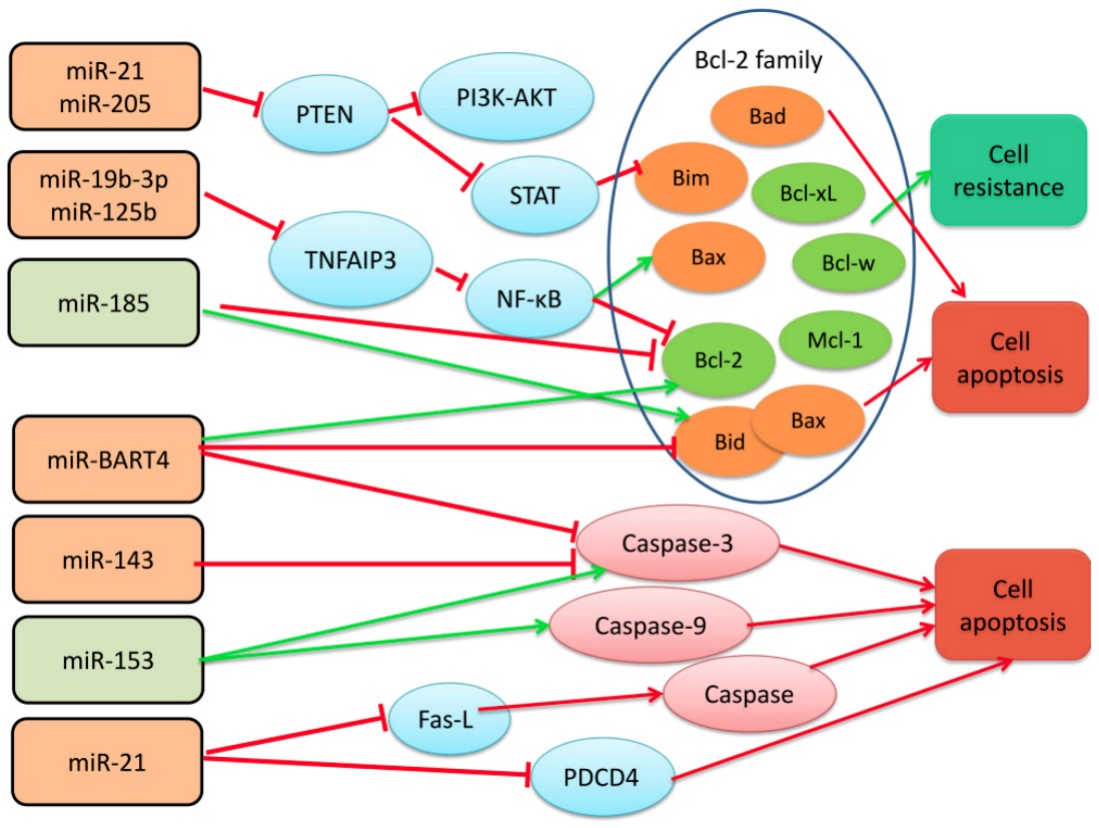
Schematic diagram of miRNAs that regulate radiotherapy resistance by Bcl-2, Caspase and Fas apoptosis families in nasopharyngeal carcinoma.

**Table 1:** miRNAs involved in NPC radioresistance

miRNA	Expression	Source	Targets or Pathway	Function	References
miR-19b-3p	up	cell and tissue	Bcl-2,Bax, Capase3, c-Capase3, TNFAIP3(A20)/ NF-κB	cell apoptosis↓	6
miR-125b	up	tissue	p-p65, γ-H2AX TNFAIP3 (A20)/ NF-κB	cell apoptosis↓	29
miR-21	up	cell and tissue	Bcl-2 , Bax, PTEN/AKT	cell survival↑, cell apoptosis↓	49, 91
miR-205	up	cell	Bcl-2 , Bax, snail, PTEN/AKT	cell survival↑, cell apoptosis↓	41
EBV-miR-BART8-3p, BART5-5p, BART7-3p, BART9-3p, BART14-3p	up	cell and tissue	γ-H2AX, ATM	DDR↑, cell apoptosis↓	31, 64, 72
miR-23a	down	cell and tissue	γH2AX, IL-8 / Stat3	DDR↓, cell cycle↓, cell apoptosis↑	77
miR-203	down	tissue	γH2AX, IL-8 / Stat3	DDR↓, cell cycle↓, cell apoptosis↑	81
miR-20a-5p	up	cell	γH2AX,Rab27B, NPAS2	cell survival↑, cell apoptosis↓	43, 44
miR-193a-3p	up	cell	SRSF2	cell cycle↓	45
miR-24	down	tissue	SP1	DDR↓	65
miR-26a	down	cell and tissue	CDK4,CDK6, EZH2	cell cycle↓	76
miR-135a	down	cell	IL-17	inflammation↓, cell apoptosis↑	82
miR-143	down	cell and tissues	capase3,PARP, γH2AX	DDR↓	87

'↑' and '↓' stand for the miRNA inhibiting or promoting the function, respectively.
